# Association Between Fever and Antibody Titer Trends After a Third Dose of the mRNA-1273 Vaccine

**DOI:** 10.2188/jea.JE20220210

**Published:** 2022-12-05

**Authors:** Naomi Matsumoto, Tomoka Kadowaki, Rumi Matsuo, Ayako Sasaki, Chikara Miyaji, Chigusa Higuchi, Masanori Nakayama, Yasue Sakurada, Hideharu Hagiya, Soshi Takao, Fumio Otsuka, Takashi Yorifuji

**Affiliations:** 1 Department of Epidemiology, Faculty of Medicine, Dentistry and Pharmaceutical Sciences, Okayama University, Okayama, Japan; 2 Department of Epidemiology, Okayama University Graduate School of Medicine, Dentistry, and Pharmaceutical Sciences, Okayama, Japan; 3 Okayama University Health Service Center, Okayama, Japan; 4 Office of Innovative Medicine, Organization for Research Strategy and Development, Okayama University, Okayama, Japan; 5 Department of General Medicine, Okayama University Graduate School of Medicine, Dentistry, and Pharmaceutical Sciences, Okayama, Japan; 6 Clinical Laboratory, Okayama University Hospital, Okayama, Japan

**Keywords:** SARS-CoV-2, mRNA-1273, antibody, reactogenicity, adverse reaction

The mRNA vaccines developed during the novel coronavirus disease 2019 (COVID-19) pandemic reportedly have a high incidence of mild-to-moderate adverse reactions, such as fever and fatigue.^1^^,^^2^ Especially mRNA-1273, one of the vaccines primarily used in Japan, tends to be eschewed because of its relatively high reported incidence of adverse reactions.^3^ Despite an outstanding clinical question, few studies have investigated whether an association exists between fever, a typical adverse reaction, and increased antibody titers after vaccination with mRNA-1273,^4^ and none have examined the association after the third dose.

Most previous studies regarding adverse reactions and antibody titers after vaccination have focused on antibody titers at only a single time point and do not provide information about titer changes over time.^5^^,^^6^ Therefore, we examined the association between the presence of fever after the third dose of mRNA-1273 and antibody titers at 1 month post-vaccination, as well as increased trends in the early post-vaccination period over time (when adverse reactions are most prevalent).

This prospective cohort study was conducted among university faculty, staff, and students who received their third dose of the mRNA-1273 vaccine at Okayama University in Japan in March 2022. A total of 49 participants agreed to participate in the study and provided written informed consent. All participants were surveyed about adverse reactions within 1 week after vaccination using Google Forms.

SARS-CoV-2 antibody titers targeting the S-protein receptor-binding domain were measured using Mokobio SARS-CoV-2 IgM & IgG Quantum Dot immunoassay^7^ with fingertip whole-blood sampling on four occasions: immediately before the third dose of mRNA-1273, at 3 days after vaccination, at 6–7 days after vaccination, and approximately 1 month after vaccination.

Following descriptive analyses, linear mixed-effects regression analysis with a random intercept and random slope was used to evaluate the different trends in antibody titers over time in the early post-vaccination period with multiple measurements according to the presence of post-vaccination fever. Poisson regression with robust variance was used to evaluate the association between post-vaccination fever and antibody titer at 1 month post-vaccination. The fixed-effects covariates in the mixed effects model examining the linear increase in antibody titer during the first week post-vaccination were the presence or absence of fever (37.5°C), sex, age group (20–49 years, 50–69 years), allergy history, antipyretic use, and an interaction term between time and each covariate, and the coefficients and 95% confidence interval (CI) were calculated. In the Poisson analysis examining the association between post-vaccination fever and elevated antibody titer (cutoff value of 25,000 IU/mL) 1 month after vaccination, we adjusted for sex, age, and allergy history (model 2), and added antipyretic analgesic use as an adjustment factor in model 3. The study protocol was approved by the Okayama University Hospital Ethics Committee (K 2112-044). All analyses were performed using Stata v.17 (StataCorp LLC, College Station, TX, USA).

Forty-seven participants (24 febrile and 23 nonfebrile) with at least two blood samplings were included in the final analysis. The febrile group was more likely to be younger and to have a history of allergies (66.7% vs 39.1% and 41.7% vs 21.7%, respectively). No participants had a known history of SARS-CoV-2 infection (eTable 1). One participant in the nonfebrile group showed an outlier value exceeding 45,000 IU/mL on the 7th day after vaccination and was excluded from all analyses because a recent SARS-CoV-2 infection was possible.

Antibody titers increased significantly faster in the febrile group than in the nonfebrile group during the first 7 days after vaccination (fixed-effect coefficient for the interaction term between fever and time [day], 960.02 [95% CI, 33.22–1886.81]) (Figure 1, eTable 2). Analysis at 1 month post-vaccination was performed in 41 patients, excluding five cases of invalid results (4 febrile and 1 nonfebrile); twelve participants (29.3%) had antibody titers above 25,000 IU/mL, and the presence of post-vaccination fever was not associated with elevated antibody titers (cutoff value of 25,000 IU/mL) 1 month post-vaccination (adjusted risk ratio 0.90; 95% CI, 0.33–2.41) (eTable 3). A sensitivity analysis using a cutoff value of 15,000 IU/mL showed similar results. In addition, no clear association between the highest body temperature and the percentage of high antibody titers (>25,000 IU/mL) 1 month post-vaccination was observed.

**Figure 1.**
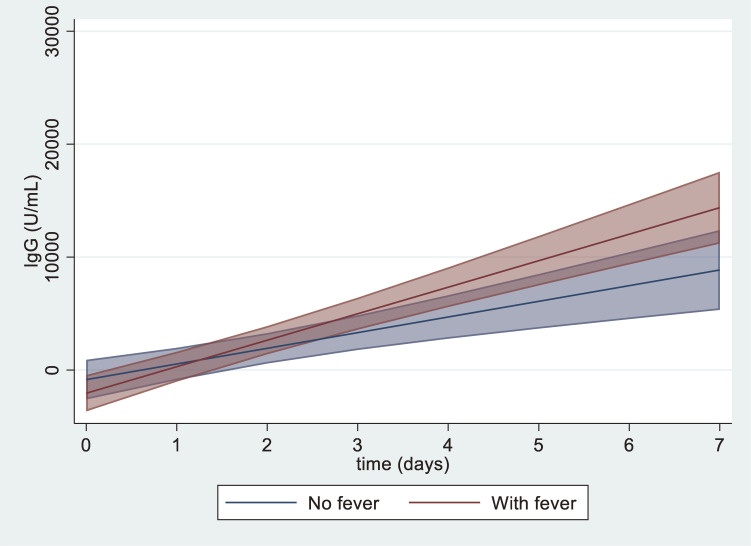
Immunoglobulin G (IgG) dynamics in the first week after vaccination by presence or absence of post-vaccination fever. The red and blue lines show the IgG concentration means predicted by the mixed-effects model in the febrile and nonfebrile groups, respectively. The area of each color indicates the 95% confidence interval (CI) of the regression curve. Day 0 is a negative value owing to the effect of the modeling prediction.

Our study had some limitations. The small sample size did not allow sufficient power to draw firm conclusions, especially for comparing antibody titers 1 month post-vaccination. Additionally, although the fingertip whole-blood test is a minimally invasive and simple method, high titers early after vaccination are often shown as invalid, and values higher than 30,000 U/mL can be inaccurate even though they are confirmed to be high. However, since the dichotomous outcome variable was used in the analysis at 1 month post-vaccination, the findings for antibody titer increases above the cutoff value are not considered to be strongly threatened. Furthermore, the five cases of invalid titers were excluded from the 1-month post-vaccination analysis, which may have underestimated the results. Despite these issues, our study suggested that the antibody titers after mRNA-1273 vaccination may be faster in the group with post-vaccination fever, but the difference may not be significant 1 month post-vaccination. The presence or absence of post-vaccination fever may not be greatly clinical meaningful. A further investigation with larger sample size is warranted.

## SUPPLEMENTARY MATERIAL

eTable 1. Characteristics of participants with and without fever after the third dose of mRNA-1273

eTable 2. Fixed-effect portion in the mixed-effects model for antibody titer trends within 1 week after vaccination (n = 46)

eTable 3. The association between post-vaccination fever and IgG titer exceeding the cutoff value of 25,000 IU/mL 1 month after vaccination (n = 41)
