# Supplementary material for: Association Between Fever and Antibody Titer Trends After a Third Dose of the mRNA-1273 Vaccine
Source: J Epidemiol. 2022 Dec 5;32(12):567–9. doi: 10.2188/jea.JE20220210 (PMC9643792; doi:10.2188/jea.JE20220210)
Supplement: Supplementary file 1 [file je-32-567-s001.pdf]

eTable 1. Characteristics of participants with and without fever after the third dose of mRNA-1273

		With fever (n=24)		Without fever (n=23)	
		n	%	n	%
Sex	female	8	33.3	8	34.8
Age, years	20–49	16	66.7	9	39.1
Underlying diseases	Yes	4	16.7	4	17.4
Allergy history	Yes	10	41.7	5	21.7
Previous SARS-CoV-2 infection	Yes	0	0.0	0	0.0
Antipyretic use	Yes	19	79.2	5	21.7

SARS-CoV-2, severe acute respiratory syndrome coronavirus 2.

eTable 2. Fixed-effect portion in the mixed-effects model for antibody titer trends within 1 week after vaccination (n=46)

Fixed effects

	Value	95% CI
(Intercept)	−1077.65	[−3726.00 to 1570.69]
Fever	−1203.45	[−3810.50 to 1403.59]
Time	1557.43	[622.77–2492.10]
Fever:time	960.02	[33.22–1886.81]
Age 50–69 years	552.36	[−1808.67 to 2913.40]
Female	−495.36	[−2873.47 to 1882.75]
Age 50–69 years:time	−644.63	[−1481.23 to 191.96]
Female:time	275.26	[−566.32 to 1116.83]
Antipyretic use	83.67	[−2611.06 to 2778.39]
Antipyretic use:time	137.96	[−816.17 to 1092.08]

CI, confidence interval.

Time was a continuous variable from days 0 to 7 after the third vaccine dose.

The symbol ":" indicates interaction.

The references for the categorical variables are as follows:

Age 20–49 years was the reference group for age 50–69 years.

Male sex was the reference group for female sex.

eTable 3. The association between post-vaccination fever and IgG titer exceeding the cutoff value of 25,000 IU/mL 1 month after vaccination (n=41)

	Model 1 (Crude model)		Model 2 (Adjusted model[a])		Model 3 (Adjusted model[b])	
	RR	95% CI	RR	95% CI	RR	95% CI
With fever	1.16	[0.44–3.03]	0.90	[0.33–2.41]	0.93	[0.33–2.59]
Age 50–69 years			0.42	[0.15–1.19]	0.42	[0.15–1.21]
Female			0.69	[0.27–1.78]	0.70	[0.27–1.81]
Allergy history			1.07	[0.38–3.06]	1.08	[0.37–3.11]
Antipyretic use					0.94	[0.34–2.63]

RR, risk ratio; CI, confidential interval.

The references for the categorical variables are as follows:

Age 20–49 was the reference group for age 50–69.

Male sex was the reference group for female sex.

[a] Adjusted for age categories, sex, and allergy history.

[b] Adjusted for age categories, sex, allergy history, and antipyretic use.
